# Functional Dissociation of Latency-Variable, Stimulus- and Response-Locked Target P3 Sub-components in Task-Switching

**DOI:** 10.3389/fnhum.2018.00060

**Published:** 2018-02-20

**Authors:** Christopher R. Brydges, Francisco Barceló

**Affiliations:** ^1^Laboratory of Neuropsychology, University of the Balearic Islands, Palma, Spain; ^2^School of Psychological Science, University of Western Australia, Crawley, WA, Australia

**Keywords:** cognitive control, event-related potentials (ERP), P300, single-trial EEG analysis, target detection, task-switching

## Abstract

Cognitive control warrants efficient task performance in dynamic and changing environments through adjustments in executive attention, stimulus and response selection. The well-known P300 component of the human event-related potential (ERP) has long been proposed to index “context-updating”—critical for cognitive control—in simple target detection tasks. However, task switching ERP studies have revealed both target P3 (300–350 ms) and later sustained P3-like potentials (400–1,200 ms) to first targets ensuing transition cues, although it remains unclear whether these target P3-like potentials also reflect context updating operations. To address this question, we applied novel single-trial EEG analyses—residue iteration decomposition (RIDE)—in order to disentangle target P3 sub-components in a sample of 22 young adults while they either repeated or switched (updated) task rules. The rationale was to revise the context updating hypothesis of P300 elicitation in the light of new evidence suggesting that “the context” consists of not only the sensory units of stimulation, but also associated motor units, and intermediate low- and high-order sensorimotor units, all of which may need to be dynamically updated on a trial by trial basis. The results showed functionally distinct target P3-like potentials in stimulus-locked, response-locked, and intermediate RIDE component clusters overlying parietal and frontal regions, implying multiple functionally distinct, though temporarily overlapping context updating operations. These findings support a reformulated version of the context updating hypothesis, and reveal a rich family of distinct target P3-like sub-components during the reactive control of target detection in task-switching, plausibly indexing the complex and dynamic workings of frontoparietal cortical networks subserving cognitive control.

## Introduction

**Figure 1:**
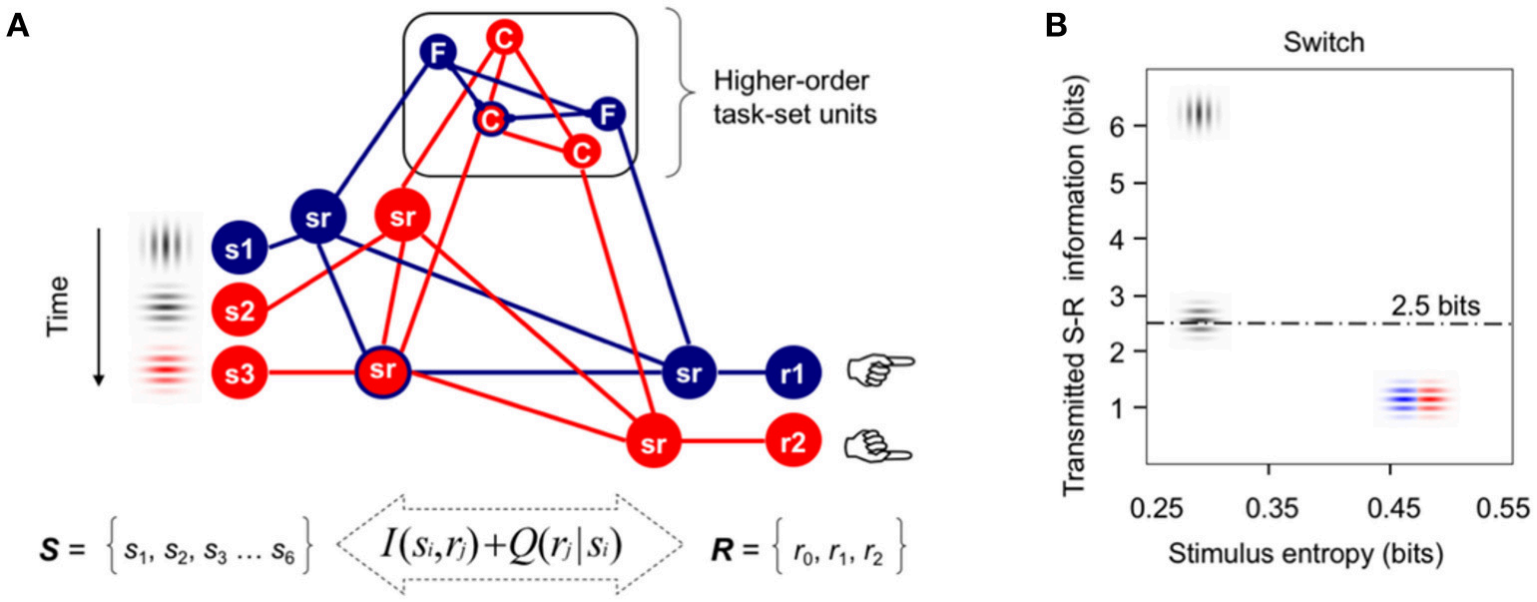
Formal modeling of task-switching demands. **(A)** Integrative model of cognitive control (adapted from Miller, 2000) used to formalize the idea that information processing demands associated with infrequent switch and repeat cues in the intermittently instructed task-cueing paradigm can be larger than those required for the reactive control of target detection and categorization. Red and blue lines indicate active higher-order task-set units and lower-order sensorimotor (*sr*) units. For simplicity, only three stimuli and two motor responses are displayed here from the pool of all available stimuli and responses necessary to categorize targets either by their Color or spatial Frequency. In this example, vertical and horizontal gray gratings are instructed as switch and repeat transition cues, respectively. **(B)** A priori estimates of transmitted S-R information between the sets of six visual stimuli and three motor responses used in our Switch task, plotted as a function of the sensory entropy of gray and color gratings. The dotted line marks the theoretical human capacity for holding information in working memory (see Supplementary Material for technical details; cf., Koechlin and Summerfield, 2007; Barceló and Cooper, 2018).

Cognitive control refers to a group of processes associated with the allocation of attentional resources in order to optimize behavioral performance whilst minimizing interference from distracting information (Botvinick et al., 2001; Gratton et al., 2018), and is associated with neural activation of a distributed frontoparietal cortical network (Niendam et al., 2012). Event-related potential (ERP) studies of cued task-switching and simpler oddball target detection tasks have both consistently reported a conspicuous P300 complex (hereafter “P3”), a positivity occurring circa 300–1,000 ms after target display (this latency window also encompasses the less well-defined Late Positive Complex, LPC; Polich, 2007)^1^, that has long been associated to “context updating” operations in working memory across different sensory modalities and many task domains (Donchin and Coles, 1988; Barceló, 2003). Further, the P3 has traditionally been conceptually and empirically split into a fronto-central P3a aspect, elicited when a temporarily unexpected or novel stimulus is presented (Friedman et al., 2001), and a centro-parietal P3b aspect, most often assumed to index the updating of the “stimulus context” (Polich, 2007). However, recent research directly comparing P3s from oddball, go/nogo and cued task switching paradigms has suggested that the sharp conceptual distinction between frontal P3a and parietal P3b potentials may be overly simplistic (Barceló and Cooper, 2018). In their study, these authors revealed two families of functionally distinct P3-like ERP positivities with roughly similar frontoparietal distributions, albeit with distinct scalp topographies and functional properties each. One of these P3 families was elicited by temporarily unpredictable cueing events, and indexed proactive control of both task and temporal uncertainty (i.e., stimulus *oddballness*). The other P3 family was elicited by temporarily predictable target events, and provided a relatively pure index of reactive control of stimulus-response selection at target onset (i.e., stimulus *targetness*). This double dissociation of frontoparietal P3-like positivities in cued task-switching was partly consistent with the original context-updating hypothesis of P300 derived from oddball target detection tasks (Donchin and Coles, 1988; Polich, 2007), although it also highlighted the importance of the temporal context (e.g., distinct proactive *vs*. reactive control modes; Braver, 2012), and of the ongoing task context (and specially, the motor and sensorimotor demands; see Figure 1A), to fully account for the richness of cue-locked P3 and target-locked P3 modulations seen across frontoparietal scalp regions (Barceló and Cooper, 2018). It remained unclear, though, to what extent proactive context-updating during the cue-target interval influenced reactive context-updating during subsequent target detection and classification, as indexed by target P3 potentials.

The present study was conceived to shed new light on the putative interaction between proactive and reactive control modes for efficient target detection, and thus, to clarify the trial-by-trial modulations of target P3 potentials observed over several target trials after switching or repeating the ongoing stimulus-response (S-R) mappings (cf., Barceló, 2003; Barceló and Cooper, 2018). In doing so, we embraced a general model of executive prefrontal function (Figure 1A; cf., Miller, 2000; Miller and Cohen, 2001) in order to reformulate the context-updating hypothesis of target P3 (Donchin and Coles, 1988) as presumably composed of a mixture of sensory, motor, as well as intermediate low- and high-order sensorimotor context updating processes, all of which may potentially take place on a trial-by-trial bases for efficient task-switching behavior. From this wider theoretical perspective, the more frontal or parietal scalp distribution of target P3 potentials may be assumed to depend on context-sensitive trial-by-trial changes in cognitive demands, as can be formally estimated with information theory metrics such as information transmission^2^ (Figure 1B; cf., Barceló et al., 2008; Barceló and Cooper, 2018). Even though the reactive control of target detection has long been equated with executive control (cf., Posner and Petersen, 1990, p. 33), now this is regarded more like a late correction mechanism invoked during detection and resolution of interference after target onset (Braver, 2012). However, interference not only is highly context-sensitive (i.e., it is normally larger following switch than repeat cues), but it can also occur at different levels along the neural hierarchy for cognitive control (either at sensory, motor, or intermediate sensorimotor levels; Figure 1A; cf., Friston et al., 2017), all of which could differentially contribute to the summated ERP waveforms that give rise to conventional target P3 potentials. Next we review the literature in support for our hypothesis of multiple context-updating component processes underlying conventional target P3 potentials, and then suggest how these can be disentangled using a novel technique for single-trial electroencephalographic (EEG) signal decomposition.

In simple oddball target detection tasks, the reactive control of target detection (Posner and Petersen, 1990, p. 33) is known to elicit a target P3b potential that has been traditionally explained as elicited when the subject's model of the environment is updated following motivationally significant events (Donchin and Coles, 1988). These traditional views define two functionally and topographically distinct P3 sub-components mostly based on the task relevance of the eliciting stimulus for goal-directed behavior. For example, according to these views “stimulus evaluation engages focal attention (P3a) to facilitate context maintenance (P3b),” and “topographic differences among potentials necessarily reflect stimulus-driven attributes” (Polich, 2007, p. 2134). Additionally, response demands have been long known to modulate P3 potentials. Thus, Falkenstein et al. (1993, 1994a,b) found early evidence of two functionally distinct P3-like positivities, P-SR and P-CR, associated with stimulus evaluation and response selection in simple and complex choice reaction tasks, respectively. The P-SR positivity showed a central scalp topography, and its latency varied with stimulus modality, whereas the P-CR positivity had a parietal topography and its latency was associated with response time (Falkenstein et al., 1993), time pressure (Falkenstein et al., 1994a), and response selection difficulty (Falkenstein et al., 1994b). More recently, Barceló and Cooper (2018) reported subtle changes in scalp topography between regular target P3b (300–350 ms) and a later sustained target LPC (400–1,100 ms) during the reactive control of task rule updating on first target trials ensuing transition cues, implying different configurations of neural sources for different target P3 sub-components as a function of cognitive demands for switching or repeating low- and high-order sensorimotor S-R links in working memory (Figure 1A). These findings pointed to the existence of, not just one, but multiple target P3-like subcomponents associated with time varying context-updating operations subserving efficient reactive control of task switching. These authors hypothesized that trial-by-trial changes in cognitive demands during reactive control of target detection translated into a whole family of target P3-like positivities recruiting multiple nodes across frontoparietal networks for cognitive control (cf., Bledowski et al., 2004; Duncan, 2010). In sum, conventional ERP analyses have long pointed to the existence of, not just one, but several distinct target-locked P3-like positivities linked to the updating of the stimulus context, the response context, as well as the context of intermediate sensorimotor low- and high-order task units within a putative hierarchy of cognitive control in the brain (Figure 1A).

However, one limitation of traditional P300 ERP research has been its inability to disentangle multiple, potentially overlapping target P3-like positivities that may be either locked to the stimulus or to the motor response, as distinct from intermediate sensorimotor operations, as all these neural processes partly overlap within the recording epoch (Luck, 2014). Hence, a target P3-like sub-component that is locked to trial-to-trial variable reaction times (RTs) is unlikely to show a clear peak, and will be smeared within the whole stimulus-locked ERP waveform. Accordingly, the sustained target P3-like positivities recorded to first targets following transition cues may reflect a mixture of functionally distinct context-updating operations associated either with the updating of stimulus attributes, of (pre)motor programs, or the updating of intermediate sensorimotor low-order S-R links and high-order task rules (Figure 1A), with more frontal scalp topographies found with increasingly larger cognitive demands involved (Figure 1B). To overcome this limitation, Ouyang et al. (2011, 2015, 2016, 2017) recently developed a novel technique for separating ERP components named “residue iteration decomposition” (RIDE), that defines clusters of ERP components based on their trial-to-trial latency variability as stimulus-locked, response-locked, and latency-variable central clusters—referred to henceforth as the S, R, and C clusters, respectively (Ouyang et al., 2017). Accordingly, the S and R clusters can be assumed to best capture context-updating operations triggered by the stimulus and response contexts, respectively, whereas the C cluster can be assumed to best index updating of intermediate sensorimotor operations (i.e., either updating to different low-order S-R mappings in repeat target trials, or else, protracted updating to different higher-order task rules in switch target trials; Figure 1A; cf., Allport et al., 1994; De Jong, 2000).

The aim of the current study was to apply novel single-trial electroencephalographic (EEG) analyses—residue iteration decomposition (RIDE; Ouyang et al., 2011, 2015, 2016, 2017)—in order to disentangle functionally distinct context-updating operations from the sustained P3-like positivities to first target trials following transition cues described by Barceló and Cooper (2018), and clarify their functional roles in cued task switching. The rationale for applying RIDE was to extend Donchin and Coles' (1988) “context updating” hypothesis in the light of new evidence suggesting that “the context” to be updated during task-switching involves not only the sensory aspects of stimulation, but also (pre)motor response units, as well as intermediate sensorimotor low- and high-order task-set units (i.e., hidden or latent variables; Friston et al., 2017), all of which may potentially need to be updated on a trial by trial basis for adaptive, goal-directed task-switching behavior (cf., Figure 1A; Barceló, 2003; Barceló et al., 2008; Barceló and Cooper, 2018).

In their study, Barceló and Cooper (2018) interpreted the sustained P3-like positivities to first target trials as reflecting early reactive control of target detection and categorization during the first implementation of a simple visuomotor rule, and they were thought to partly index working memory overload from the preceding cueing event (Figure 1B). These sustained target P3-like positivities were larger following switch compared to repeat cues, thus providing an EEG index of switch costs for first targets that matched switch costs in behavioral accuracy. In contrast, mean RTs were similarly increased in switch and repeat trials, and only captured residual restart costs given the long cue-target interval employed (cf., Monsell, 2003, 2017). In subsequent target trials, the sustained P3-like positivity decreased rapidly, and completely faded away in third target trials (i.e., after the same task rule had been rehearsed three times), where all visual targets elicited the classic target P3b (peak latency 350–400 ms) showing similar amplitudes and mid-parietal scalp distributions regardless of the meaning of the previous cue for switching or repeating rules. These findings highlighted the trial-by-trial dynamics of “context-updating” operations underlying elicitation of target P3-like positivities during reactive control of target detection (Barceló and Cooper, 2018). However, this study did not clarify what type of contextual information (sensory, motor, or sensorimotor) indexed the sustained target P3-like positivities to first targets, or how these mapped onto behavioral indexes of task-switching efficiency.

The current study aimed to further explore the nature of sustained target P3-like positivities to first target trials originally reported by Barceló and Cooper (2018), by employing RIDE-decomposed latency-locked (i.e., stimulus- and response-locked) and latency-variable (i.e., central, cognitive) clusters. Inspired on a reformulated version of the context updating hypothesis (Donchin and Coles, 1988; Miller and Cohen, 2001; Polich, 2007), it was postulated that sustained target P3-like positivities could be elicited not only by the updating the sensory context (stimulus-locked), but also by the updating of motor and/or premotor (response-locked) units, as well as low- and higher-order sensorimotor (i.e., cognitive) task-set units along the putative neural hierarchy of cognitive control (Figure 1A; cf., Barcelo and Knight, 2007; Barceló et al., 2008; Friston et al., 2017). Further, and regardless of the nature of the contextual information being updated, first target trials following a switch cue were predicted to demand higher cognitive control than first repeat target trials, or than any third targets in the trial sequence (Figure 1B). From these premises, three hypotheses were formulated. First, it was hypothesized that the largest differences in the amplitude of target P3-like positivities elicited during task rule updating (switch *vs*. repeat) and target trial (target 1 vs. 3 after a transition cue) would be reflected mostly in the C cluster, which indexes latency-variable cognitive operations (i.e., carry-over of interference from a previous S-R mapping, and/or protracted task-set reconfiguration to high-order task-set units; Allport et al., 1994; De Jong, 2000), with more frontal scalp topographies predicted for those conditions with larger cognitive demands. That is, more frontal target P3-like positivities were predicted for first target trials following a switch than a repeat cue, with less frontal P3-like positivities predicted once the same rule was rehearsed three times in a row. Second, the S cluster was predicted to evoke target P3-like activity triggered by the updating of the sensory context, with an expected centro-parietal scalp distribution for visual stimulation. Besides, since all visual targets were temporarily predictable and frequent events (each *p* = 0.21) that shared similar sensory features in all task conditions, no differences in mean target P3 amplitudes for this S cluster were expected across task conditions. Third, context-updating was also assumed to be triggered by trial-by-trial changes in (pre)motor processes (i.e., the R cluster), as response button presses are constantly updated on a trial-by-trial bases (Figure 1A; cf., Falkenstein et al., 1994b; Barceló and Cooper, 2018). To the best of the authors' knowledge, no study has so far explored the contribution from the R cluster to target P3 elicitation. In principle, and given the similar response demands for all targets, no differences in mean P3 amplitudes from the R cluster were expected across task conditions.

## Materials and methods

### Participants

Twenty-two young adults who were students at the University of the Balearic Islands (three males, *M* = 21.6 years, *SD* = 2.6 years) participated in the study (this is a subset of the same participants examined by Barceló and Cooper (2018), being used here to explore new target P3 hypotheses using the RIDE technique). All participants had normal or corrected-to-normal vision and reported no history of neurological or psychiatric disorders. Informed consent was obtained from all participants and experimental procedures and behavioral testing was performed in accordance with the Declaration of Helsinki, and with the approval of the Ethics committee of the university. All participants had accuracy greater than 80% to ensure that only highly efficient participants entered the final sample.

### Materials

**Figure 2:**
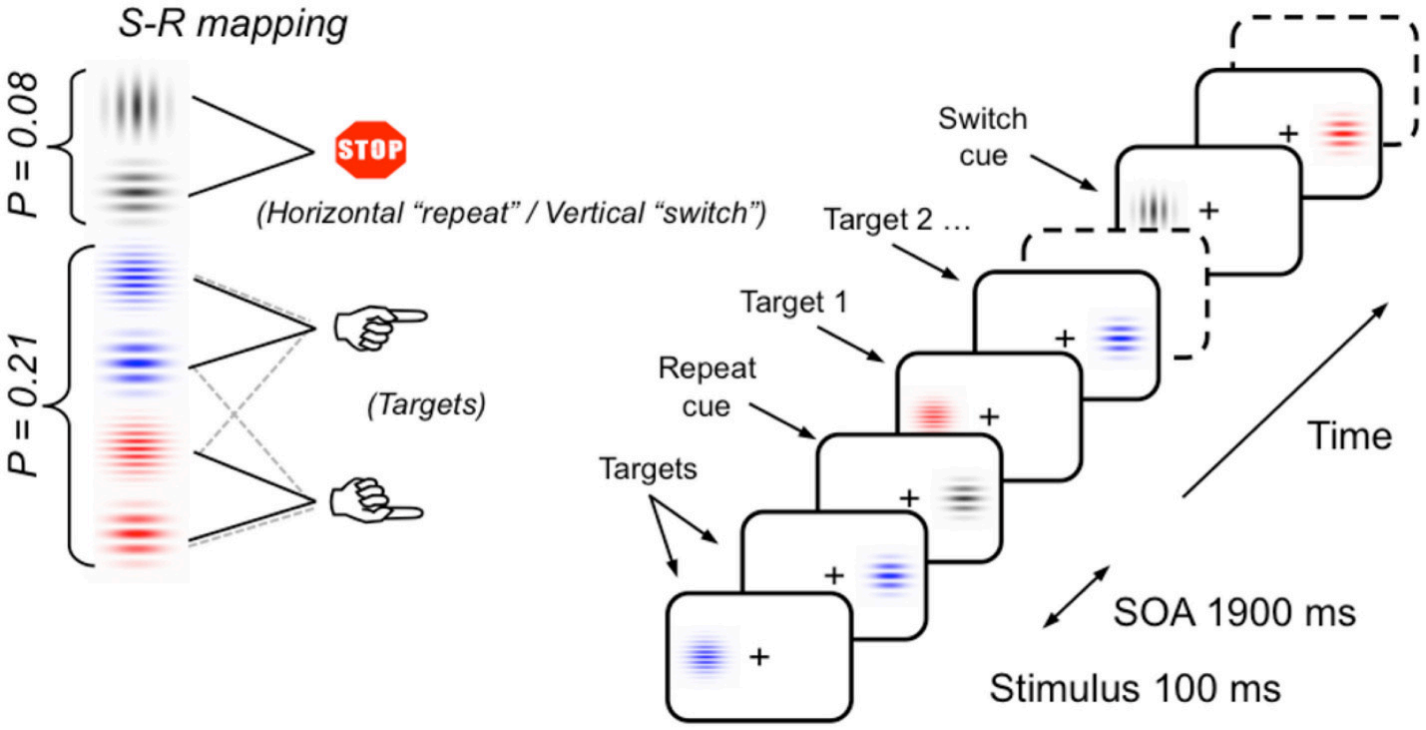
Schematic of the task-switching paradigm, stimulus materials and instructed S-R mappings. Infrequent vertical and horizontal gray gratings intermittently cued participants to switch and repeat the previous S-R mapping (i.e., sorting the frequent colored gratings by their color or their thickness), respectively. In half the participants, the meaning of gray gratings orientation for switching or repeating the previous sorting rule was reversed (see section Materials and Methods for a full description; cf., Barceló and Cooper, 2018).

The same task-switching paradigm as used by Cooper et al. (2016) and Barceló and Cooper (2018) was used in the current study (see Figure 2). Participants sat in a sound attenuated and dimly lit room 150 cm from a 27 inch video LCD monitor (800 × 600 at 75 Hz). Stimuli were displayed against a dark gray background either side of a central fixation cross. Stimuli consisted of four equally probable (*p* = 0.21) colored Gabor gratings with horizontally oriented gratings (either red or blue, 4 or 10 cpd, 25% contrast, 1° visual angle, 3.5 cd/m^2^), and two infrequent (*p* = 0.08) gray Gabor gratings (oriented either vertically or horizontally, 2 cpd, 25% contrast, 1° visual angle, 3.5 cd/m^2^). Participants responded via a handheld response pad with their left or right index finger. Stimulus material was chosen to keep memory demands and novelty effects to a minimum.

The switch task was a variant of the intermittent-instruction paradigm (Monsell, 2003). The gray Gabor stimuli were the transition cues, indicating whether to switch or repeat task. The colored Gabor stimuli were the targets and required a left or right hand response based on either the color (blue or red grating) or the grating's spatial frequency (thick or thin grating). Hence, the vertical or horizontal orientation of gray Gabor gratings (transition cues) instructed participants whether to switch or repeat the task they completed on the previous trial run. The relation between gray grating orientation and instruction was counterbalanced between participants. Before the switch task, a short block of 74 test trials was administered to ensure that participants understood task instructions. The task consisted of 976 trials (divided into eight blocks) of colored and gray Gabor gratings, the order of which was semi-randomly generated offline, with the constraint that two consecutive gray Gabor gratings were separated by four to eight colored gratings. Each trial consisted of a Gabor grating presented for 100 ms in the left or the right visual hemifield. On target trials, participants had to respond within a maximum of 1,200 ms after stimulus onset. Participants were instructed to fixate their gaze on the central cross and avoid shifting their eye gaze to the lateralized Gabor stimuli. Instructions emphasized both response speed and accuracy. All error trials (i.e., incorrect, late responses and false alarms to gray gratings) were followed by visual feedback and the following trial was delayed by 500 ms to help subjects keep on task. Hence, interstimulus intervals were either 1,900 or 2,400 ms for correct and incorrect trials, respectively. The stimulus display and behavioral response recording were carried out using Presentation® software (Neurobehavioral Systems Inc., Albany, CA).

### Behavioral analyses

Correct trial runs were defined as those containing no errors on the first three target trials following a task cue. Reaction times (RTs) are reported from correct trial runs only, and errors committed on the first three trials were used to compute accuracy indexes. Only the first three target trials following a gray grating entered the analyses, since behavioral costs typically reach an asymptote in later trials (Monsell, 2003). Restart costs were calculated by subtracting the mean RTs of target 3 from target 1 for each participant, separately for both switch and repeat task conditions. Local switch costs were calculated by subtracting the mean RT of repeat target 1 from switch target 1 for each participant. However, no switch-specific behavioral local cost was expected given our long cue-target intervals (1,900 ms), and since advanced task-set reconfiguration of simple task rules is normally fully completed in less than one second in correct trials (Monsell, 2003). Mean RTs and percentage error trials were subjected to repeated measures analysis of variance (ANOVA) with rule updating (switch vs. repeat) and target trial (target 1 vs. 3 in the trial run; T1 vs. T3 for short) as the factors.

### EEG recording and processing

The electroencephalogram (EEG) was continuously recorded (0.05–100 Hz bandpass) using SynAmps RT amplifiers (NeuroScan, TX, USA) at a sampling rate of 500 Hz. Electrodes were placed at 62 scalp sites mounted on an elastic cap (Synamp2 Quikcap, Compumedics, TX). EEG electrodes were placed following the extended 10–20 position system (Fp1, Fpz, Fp2, AF7, AF3, AFz, AF4, AF8, F7, F5, F3, F1, Fz, F2, F4, F6, F8, FT7, FC5, FC3, FC1, FCz, FC2, FC4, FC6, FT8, T7, C5, C3, C1, Cz, C2, C4, C6, T8, TP7, CP5, CP3, CP1, CPz, CP2, CP4, CP6, TP8, P7, P5, P3, P1, Pz, P2, P4, P6, P8, PO7, PO3, POz, PO4, PO8, O1, Oz, O2, Iz). During recording, the left mastoid was set as reference. Four additional electrodes were placed above and below the left eye and on the outer canthi of both eyes to monitor blinks and eye movements. Prior to recording, impedances were below 10 kΩ.

EEG data were processed using MATLAB (Mathworks, Navick, MA) through a pipeline utilizing EEGLAB version 14.0.0 (Delorme and Makeig, 2004), ERPLAB version 6.1.3 (Lopez-Calderon and Luck, 2014) and ADJUST version 1.1.1 (Mognon et al., 2011). Preprocessing was performed in EEGLAB by decreasing the sampling rate to 250 Hz, re-referencing offline to linked mastoids, and bandpass filtering the data (0.1–30 Hz). Epochs for each stimulus type were extracted from 200 ms prestimulus to 1,200 ms poststimulus onset. Independent components analysis was conducted using the extended Infomax algorithm (Bell and Sejnowski, 1995), and the ADJUST toolbox was used to detect any artifactual components (including blinks, eye movements and muscle movement). These components were removed, and the remaining components were back-projected to the electrode space. Epochs with residual ocular or muscular artifacts were manually removed. Further, to ensure high quality data for the RIDE analyses, epochs containing EEG signals exceeding ±100 μV at any electrode site were excluded from analysis, and only participants with a minimum of 30 clean epochs per task condition entered the RIDE analyses (Ouyang et al., 2015). These strict data requirements motivated the exclusion of *n* = 9 participants from the original dataset (cf., Cooper et al., 2016; Barceló and Cooper, 2018). The average number of epochs per participant was 60.95 (*SD* = 7.41) each for switch target trials 1 and 3, and *M* = 63.55 (*SD* = 8.86) each for repeat target trials 1 and 3.

### Electrophysiological analyses

Only correct trial runs entered the EEG analyses, while trial runs containing any false alarm, omission, or other errors on the three first target trials after a task cue were discarded. For each individual participant, ERPs extracted from switch and repeat target trials 1 and 3 (i.e., the first and third targets following the switch or repeat cue) were analyzed. Target trial 2 ERPs were not analyzed in order to maximize trial-by-trial differences in EEG/ERP activity. Specifically, any cognitive control processes associated with task switching were expected to be maximal on target 1 and minimal on target 3, with target trial 2 reflecting a mixed intermediate stage (cf., Barceló and Cooper, 2018). All analyses were conducted at the Fz and Pz electrodes in order to examine these processes across both frontal and parietal regions. Only these two electrodes were analyzed in order to simplify the statistical design and to match the extant target P3 literature.

### Residue iteration decomposition (RIDE)

The RIDE analysis followed the methods described in Ouyang et al. (2011, 2015). The RIDE toolbox and manual can be found at http://cns.hkbu.edu.hk/RIDE.htm. This technique decomposes the ERP waveform into stimulus-locked, response-locked, and central clusters (S-, R-, and C-clusters, respectively). The latency estimates of S and R are the stimulus onset and response time, respectively. The latency estimate of component cluster C is derived from the data of each individual participant using the iterative process described below. RIDE assumes that the C cluster is neither stimulus- nor response-locked, and the C cluster latency is variable over single trials as a result of this. Hence, the C cluster is a good candidate to capture the inter-subject and inter-trial variability of higher-order cognitive control assumed to be involved in resolving interference from a previous S-R mapping, and/or during delayed reconfiguration to high-order task-set units (Allport and Wylie, 2000; De Jong, 2000; Karayanidis et al., 2011). That is, depending on each trial and subject, task-set reconfiguration (aka, task rule updating) processes may take place during the cue-target interval, and partly also spill over the onset of the first, or even subsequent targets (Allport et al., 1994; De Jong, 2000). Therefore, RIDE separates component clusters by combining methods examining inter-component latency variability and single-trial latency estimation. The decomposition module makes use of both external time markers (e.g., stimulus and response onset) and estimated component latencies. The latency-locked S and R clusters are removed from the single-trial data before the latency-variable component C cluster is estimated (cf., Ouyang et al., 2011, 2015).

### Statistical analyses

**Figure 3:**
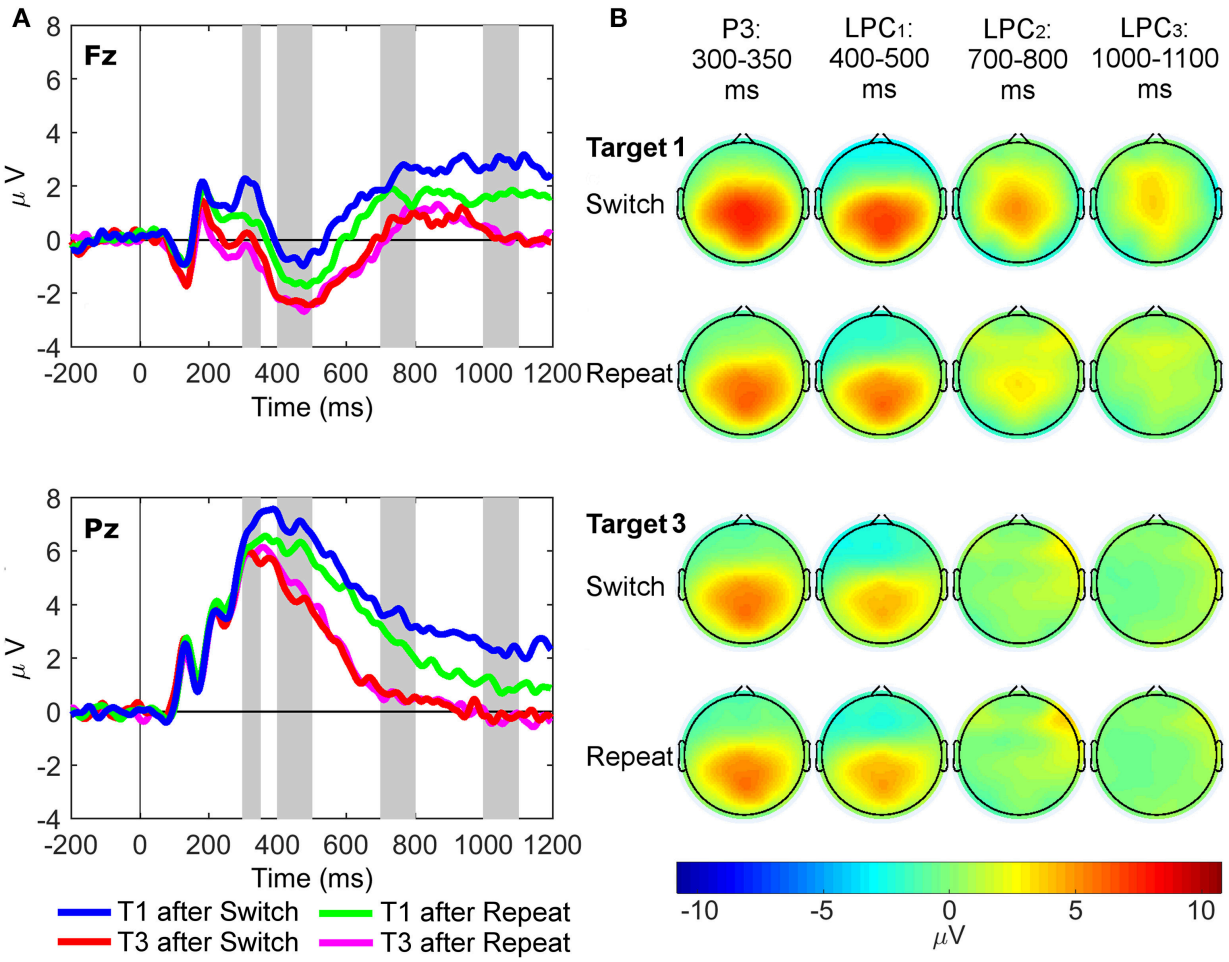
Stimulus-locked grand average ERP waveforms and scalp topography maps. **(A)** Waveforms depict mean voltages recorded from Fz (top) and Pz (bottom) electrode sites. Shaded areas indicate time windows used to measure mean ERP amplitudes tracking the temporal dynamics of the target P3-like complex: P3 (300–350 ms), LPC_1_ (400–500 ms), LPC_2_ (700–800 ms), and LPC_3_ (1,000–1,100 ms). T1: First target in the trial run, T3: Third target in the trial run. **(B)** Scalp topographies of the four late target P3-like positivities depicted in **(A)** across task conditions (i.e., first and third targets in trial runs starting either with a switch or a repeat cue).

Three-way repeated measures ANOVAs were conducted with site (Fz and Pz), rule updating (switch and repeat) and trial (target 1 and 3) as factors, with paired-samples *t*-tests used for simple tests of effects. Mean amplitudes of the grand average ERP waveforms from 300 to 350 ms (regular target P3 component), 400 to 500 ms (LPC_1_), 700 to 800 ms (LPC_2_), and 1,000 to 1,100 ms (LPC_3_) post-stimulus onset were used in the ERP analyses. These latency windows were based on previous target P3 research (e.g., Polich, 2007; Barceló and Cooper, 2018), and on visual inspection of the grand-mean ERP waveforms to first switch target trials (Figure 3), as this task condition most clearly showed the sustained switch positivity that we wanted to examine further using the new RIDE technique. The latency of the P300 is known to vary as a function of task difficulty (Kappenman and Luck, 2012), so target P3-like positivities in the current study were extracted from a wide (300–1,100 ms) latency window, and our analyses examined differences in amplitude and scalp topography, rather than any latency differences, consistent with Barceló and Cooper (2018). Likewise, RIDE analyses for the S and C clusters were also based on these same latency windows in order to examine corresponding target P3-like positivities in the S cluster (henceforth referred to as sP3 subcomponent), and the C cluster (cP3, cLPC_1_, cLPC_2_, and cLPC_3_ subcomponents). Additionally, effects of trial-by-trial motor response updating were examined on mean amplitudes of the R cluster, measured from 50 ms pre-response to 50 ms post-response at the Pz site only (rP3). Further frontal pre- and post-response P3-like peaks were also observed in the R cluster at Fz only (referred to as frontal pre-rP3 and post-rP3, respectively), whose mean amplitudes were calculated from 50 ms pre-peak amplitude to 50 ms post-peak amplitude at Fz only.

In addition to traditional null hypothesis significance testing, we included Bayesian methods. Bayesian statistics are advantageous over conventional frequentist statistics for a number of reasons (Wagenmakers et al., 2018). First, Bayesian hypothesis testing allows us to accept or reject a hypothesis by gathering evidence in favor of it, and thus, the alternative hypothesis can only be falsified by accepting the null hypothesis over it (Dienes, 2011; Kruschke, 2013). Second, Bayesian statistics allow researchers to repeatedly test the same data points without pre-committing to a specified sample size, whereas this cannot be easily done with frequentist statistics (Wagenmakers et al., 2018). Third, Bayesian statistics are produced in terms of the probability of hypotheses given data, not of data given hypotheses (Cohen, 1994), and as such, they are more interpretable than frequentist statistics to assess the credibility of one hypothesis over another (Dienes, 2011; Kruschke, 2013; Wagenmakers et al., 2018). Hence, Bayesian methods are specially well suited for testing hypotheses about potentially different target P3-like positivities using repeated measurements and analyses, and specially in order to counteract potential Type I errors and bogus findings associated with *p*-values of conventional frequentist statistics (Luck and Gaspelin, 2017).

For the traditional ERP and the RIDE analyses, repeated-measures ANOVAs were calculated to test whether ERP/cluster amplitudes were affected by task rule updating and target trial. A Bayes Factor (BF) was calculated from the ANOVA to test how much the data supported the alternative (H_1_) over the null (H_0_) hypothesis. Based on guidelines set by Jeffreys (1961), a BF_10_ > 3 was considered sufficient evidence in favor of the alternative hypothesis, and a BF_10_ > 10 was considered to be strong evidence in favor. Of note, BF_10_ refers to the BF value of H_1_ being supported over H_0_, whereas BF_01_ refers to the opposite. To calculate BF_01_, one simply inverts the BF_10_ value. Additionally, posterior probabilities were calculated (the probability of a hypothesis being correct given the observed data; Masson, 2011). Alternative hypotheses would only be accepted if the ANOVA was statistically significant in the expected direction, and BF_10_ > 3. These BF values were calculated using the MATLAB toolboxes EEGLAB and ERPLAB, as well as custom-made MATLAB scripts based on the formulae described by Jarosz and Wiley (2014). For correlations between behavioral measures (RTs, accuracy, and switch costs) and RIDE cluster amplitudes, Pearson's correlations and Bayes factors were calculated using JASP version 0.8.1.2 (JASP Team, 2017).

## Results

### Behavioral results

Participants performed the task at a high level, with a mean accuracy of 90.8% (*SD* = 4.1%) for target 1 and 92.6% (*SD* = 3.5%) for target 3. A 2 × 2 (rule updating x target trial) repeated measures ANOVA was conducted on mean RTs. Although the interaction failed significance [*F*_(1, 21)_ = 2.84, *p* = 0.11, $\eta_{\text{p}}^{2}$ = 0.12], the main effects for target trial [*F*_(1, 21)_ = 25.63, *p* < 0.001, $\eta_{\text{p}}^{2}$ = 0.55] and rule updating [*F*_(1, 21)_ = 10.68, *p* = 0.004, $\eta_{\text{p}}^{2}$ = 0.34] both were significant, with mean RTs being significantly longer for target 1 than for target 3 trials (see Table 1).

**Table 1:** Mean RTs (SD) and residual (restart, switch) costs.

**Target trial**	**Switch**	**Repeat**	**Switch costs**
Target 1 (T1)	508.8 (89.1)	538.6 (89.8)	−29.8 (49.7)
Target 3 (T3)	491.4 (83.8)	502.3 (79.6)	–
Restart costs	17.5 (41.1)	36.3 (30.4)	

When examining costs, switch targets showed marginally significant restart costs [*t*_(21)_ = 1.99, *p* = 0.059, Cohen's *d* = 0.20], while repeat targets showed highly significant restart costs [*t*_(21)_ = 5.60, *p* < 0.001, Cohen's *d* = 0.43]. There was a switch benefit on first target trials [*t*_(21)_ = 2.79, *p* = 0.011, Cohen's *d* = 0.60], which probably reflects residual switch costs, as reported in previous task switching studies using long cue-target intervals (e.g., Forstmann et al., 2007; Periáñez and Barceló, 2009; Monsell, 2017; Díaz-Blancat et al., 2018).

### Conventional ERP results

Figure 3 shows grand average waveforms and scalp maps of target P3-like positivities to first and third targets immediately following switch and repeat cues, respectively.

#### Target P3 (300–350 ms)

Two significant two-way interactions between site and rule updating [*F*_(1, 21)_ = 4.50, *p* = 0.046, $\eta_{\text{p}}^{2}$ = 0.18, BF_10_ = 5.13, posterior probability = 0.84], and site and target trial [*F*_(1, 21)_ = 7.45, *p* = 0.013, $\eta_{\text{p}}^{2}$ = 0.26, BF_10_ = 17.14, posterior probability = 0.94] revealed that P3 amplitudes were larger for switch than repeat trials at both Fz [*t*_(21)_ = 2.91, *p* = 0.008] and Pz [*t*_(21)_ = 2.18, *p* = 0.041]. Likewise, target P3 amplitudes were larger for target 1 than target 3 trials at both sites [main effect of target trial *F*_(1, 21)_ = 19.87, *p* < 0.001, $\eta_{\text{p}}^{2}$ = 0.49, BF_10_ = 919.61, posterior probability > 0.99], although these trial differences were significantly larger at Fz [*t*_(21)_ = 5.07, *p* < 0.001] than at Pz [*t*_(21)_ = 2.35, *p* = 0.028]. Significant main effects were found for site [Pz > Fz; *F*_(1, 21)_ = 68.41, *p* < 0.001, $\eta_{\text{p}}^{2}$ = 0.77, BF_10_ = 5.00 × 10^6^, posterior probability > 0.99] and target trial [T1> T3; *F*_(1, 21)_ = 8.93, *p* = 0.007, $\eta_{\text{p}}^{2}$ = 0.30, BF_10_ = 29.93, posterior probability = 0.97].

#### Target LPC_1_ (400–500 ms)

The interaction between rule updating and target trial was significant [*F*_(1, 21)_ = 6.11, *p* = 0.022, $\eta_{\text{p}}^{2}$ = 0.23, BF_10_ = 10.06, posterior probability = 0.91], revealing larger amplitudes for switch than repeat trials for target 1, [*t*_(21)_ = 2.53, *p* = 0.020]. There were significant main effects of site [Pz > Fz; *F*_(1, 21)_ = 121.10, *p* < 0.001, $\eta_{\text{p}}^{2}$ = 0.85, BF_10_ = 8.25 × 10^6^, posterior probability > 0.99] and target trial [T1 > T3; *F*_(1, 21)_ = 47.47, *p* < 0.001, $\eta_{\text{p}}^{2}$ = 0.69, BF_10_ = 2.68 × 10^5^, posterior probability > 0.99].

#### Target LPC_2_ (700–800 ms)

Only the main effect of target trial reached significance [T1 > T3; *F*_(1, 21)_ = 37.59, *p* < 0.001, $\eta_{\text{p}}^{2}$ = 0.64, BF_10_ = 4.80 × 10^4^, posterior probability > 0.99].

#### Target LPC_3_ (1,000–1,100 ms)

A significant interaction between rule updating and target trial [*F*_(1, 21)_ = 9.88, *p* = 0.005, $\eta_{\text{p}}^{2}$ = 0.32, BF_10_ = 42.09, posterior probability = 0.98] demonstrated larger amplitudes for switch than repeat trials on target 1 [*t*_(21)_ = 3.54, *p* = 0.002], with no differences between switch and repeat conditions in target 3 [*t*_(21)_ = −0.03, *p* = 0.98]. The main effects of rule updating [Switch > Repeat; *F*_(1, 21)_ = 8.40, *p* = 0.009, $\eta_{\text{p}}^{2}$ = 0.29, BF_10_ = 24.52, posterior probability = 0.96] and target trial [T1 > T3; *F*_(1, 21)_ = 42.99, *p* < 0.001, $\eta_{\text{p}}^{2}$ = 0.67, BF_10_ = 1.27 × 10^5^, posterior probability > 0.99] also reached significance.

In sum, conventional ERP analyses indicated that both rule updating and target trial yielded significant effects starting at the target P3 latency window, and were also seen at later first target LPC windows, suggesting additional frontal resources were recruited to process first target trials immediately following both switch and repeat cues.

### RIDE results

**Figure 4:**
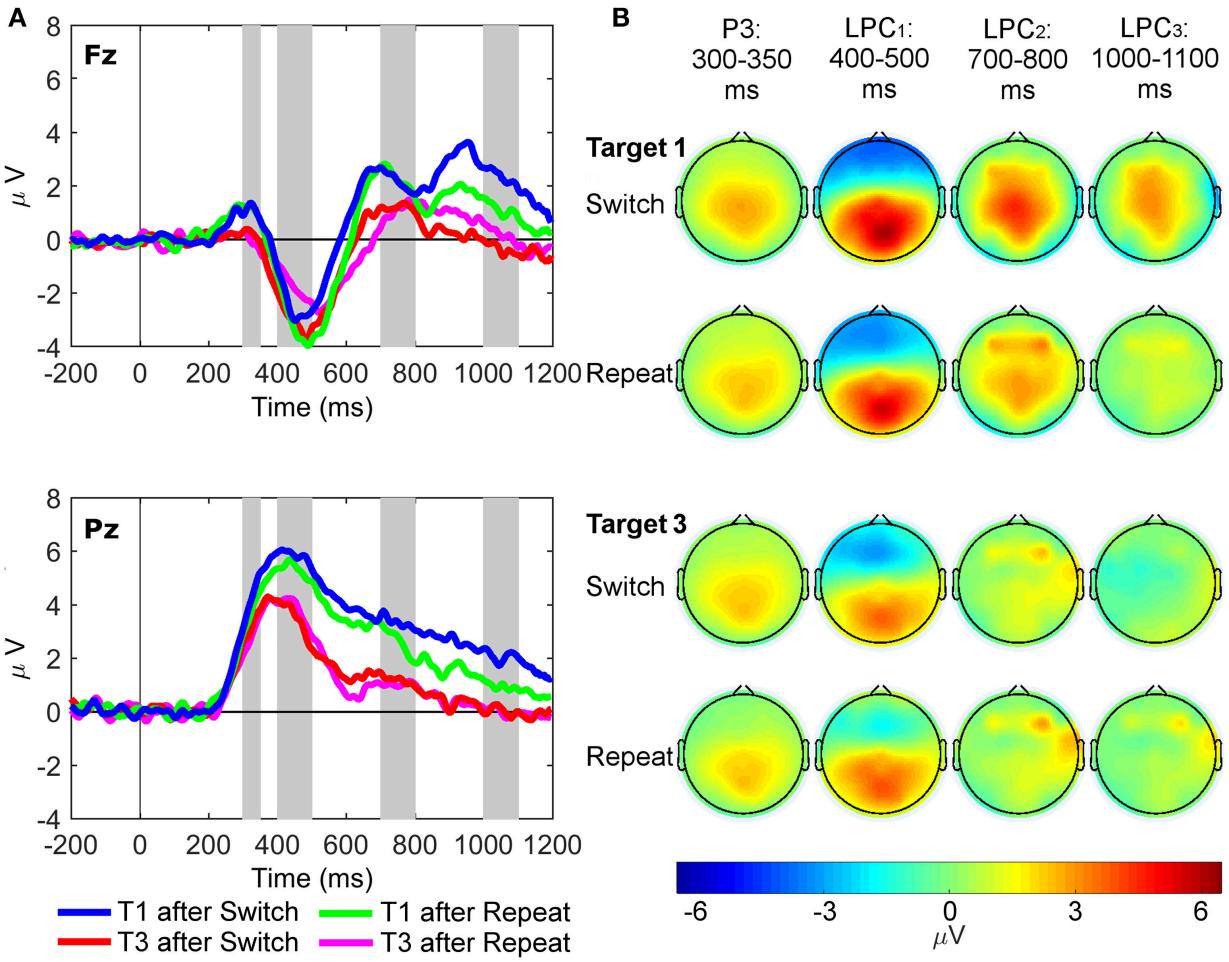
Latency-variable C cluster waveforms and scalp topography maps. **(A)** Waveforms depict mean voltages recorded from Fz (top) and Pz (bottom) electrode sites. Shaded areas indicate time windows used to measure mean amplitudes tracking the temporal dynamics of the C cluster target P3-like complex: cP3 (300–350 ms), cLPC_1_ (400–500 ms), cLPC_2_ (700–800 ms), and cLPC_3_ (1,000–1,100 ms). T1: First target in the trial run, T3: Third target in the trial run. **(B)** Scalp topographies of the four cP3-like positivities depicted in **(A)** across task conditions (i.e., first and third targets in trial runs starting either with a switch or a repeat cue).

**Figure 5:**
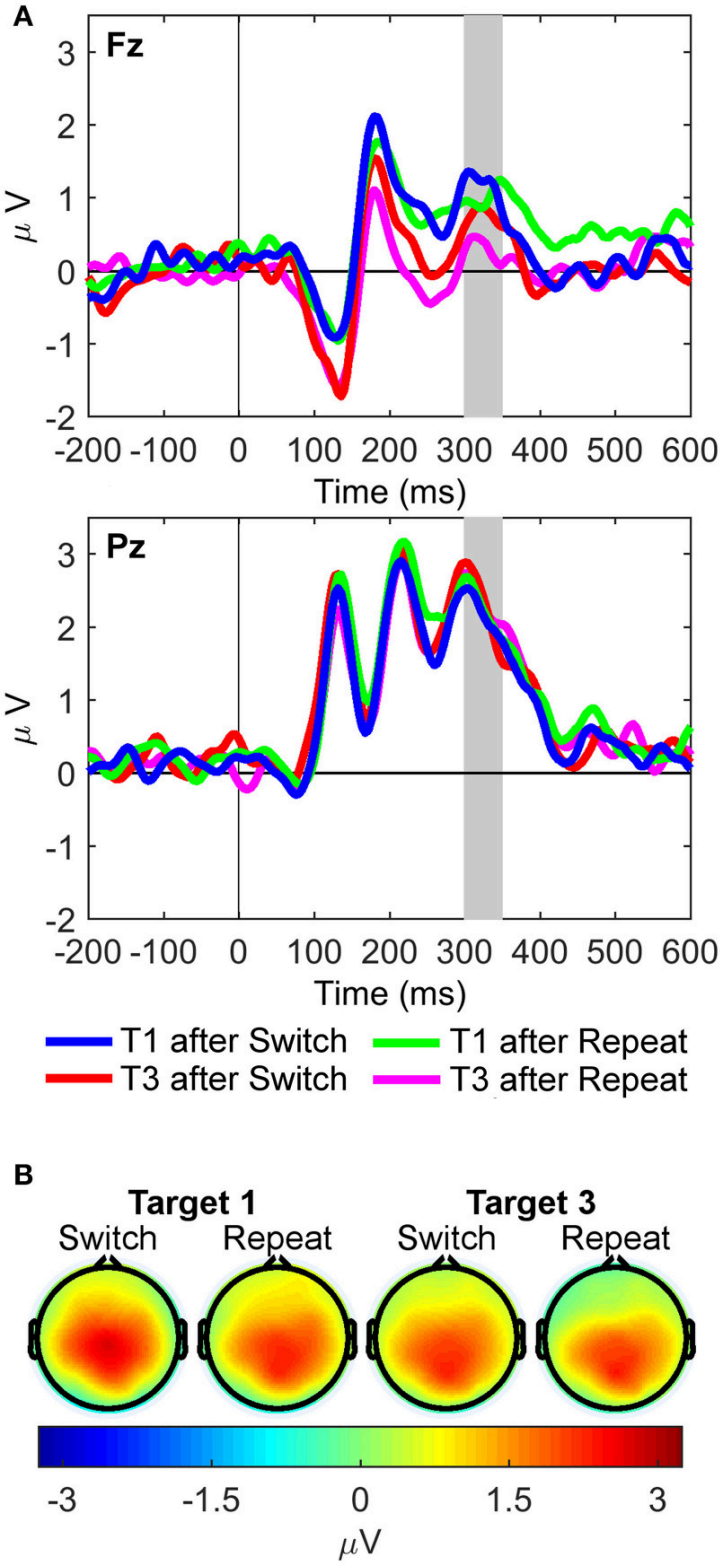
Stimulus-locked waveforms and scalp maps for the S cluster. **(A)** Waveforms depict grand-averages recorded from Fz (top) and Pz (bottom). The shaded area is the latency window used to measure P3-like activity in the S cluster: sP3 (300–350 ms). **(B)** Scalp topographies for each task condition are mean amplitudes within the shaded time window in the waveforms.

**Figure 6:**
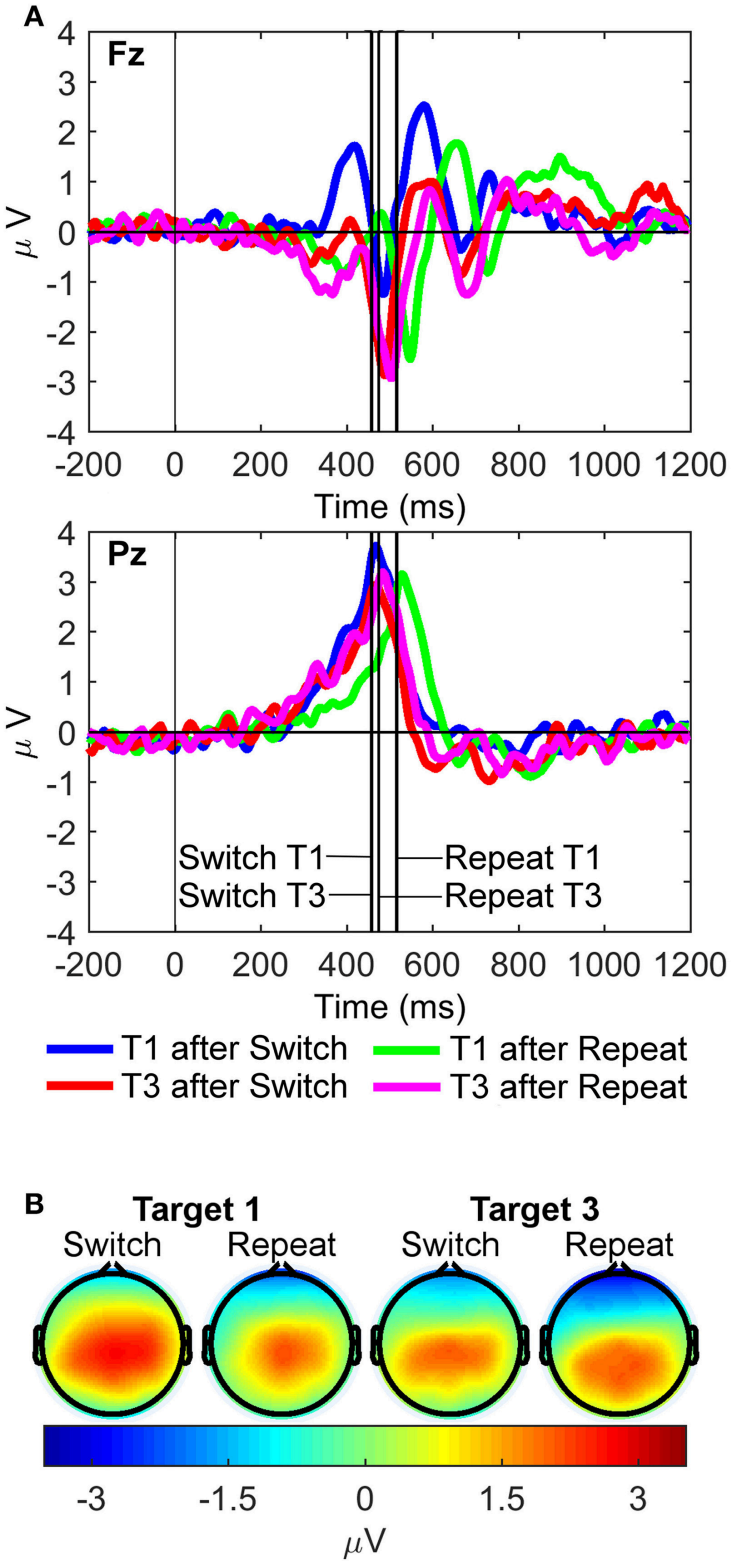
Response-locked waveforms and scalp maps for the R cluster. **(A)** Waveforms depict grand-averages recorded from Fz (top) and Pz (bottom). Vertical lines indicate the median response time for each task condition. **(B)** Scalp topographies for each task condition are the mean amplitudes measured in a 50 ms pre-response to 50 ms post response time window around the median response time for each condition.

Waveforms and scalp maps for the C, S, and R clusters are displayed in Figures 4–**6**, respectively. The C cluster consisted of both a target P3-like (cP3; 300–350 ms) component and a late positive complex (cLPC; 400–1,200 ms) overlying both frontal and parietal regions, which mimicked those observed in the conventional ERP waveforms. The S cluster consisted not only of early sensory potentials (90–150 ms), but also later latency P2-like (180–230 ms) and target P3-like (sP3; 300–350 ms) positivities. Finally, the R cluster showed a target P3-like component (rP3) with maximal amplitude over parietal regions at the median response time of each task condition.

#### C cluster

For the cP3 component (Figure 4), there were significant main effects of site [Pz > Fz; *F*_(1, 21)_ = 61.93, *p* < 0.001, $\eta_{\text{p}}^{2}$ = 0.75, BF_10_ = 2.20 × 10^6^, posterior probability > 0.99] and target trial [T1 > T3; *F*_(1, 21)_ = 25.50, *p* < 0.001, $\eta_{\text{p}}^{2}$ = 0.55, BF_10_ = 3.81 × 10^3^, posterior probability >0.99]. For the cLPC_1_ component, two-way interactions between site and target trial [*F*_(1, 21)_ = 11.97, *p* = 0.002, $\eta_{\text{p}}^{2}$ = 0.36, BF_10_ = 86.62, posterior probability = 0.99] and rule updating and target trial were significant [*F*_(1, 21)_ = 7.14, *p* = 0.014, $\eta_{\text{p}}^{2}$ = 0.25, BF_10_ = 15.18, posterior probability = 0.94]. *Post-hoc* tests showed larger cLPC_1_ amplitudes for first than third switch trials only at Pz [*t*_(21)_ = 5.61, *p* < 0.001]. There were significant main effects of site [Pz > Fz; *F*_(1, 21)_ = 132.25, *p* < 0.001, $\eta_{\text{p}}^{2}$ = 0.86, BF_10_ = 1.00 × 10^9^, posterior probability > 0.99] and target trial [T1 > T3; *F*_(1, 21)_ = 11.43, *p* = 0.003, $\eta_{\text{p}}^{2}$ = 0.35, BF_10_ = 72.26, posterior probability = 0.99]. The cLPC_2_ component only showed a main effect of target trial [T1 > T3; *F*_(1, 21)_ = 21.83, *p* < 0.001, $\eta_{\text{p}}^{2}$ = 0.51, BF_10_ > 1,500, posterior probability > 0.99]. Finally, the cLPC_3_ component showed a significant two-way interaction between rule updating and target trial [*F*_(1, 21)_ = 16.76, *p* < 0.001, $\eta_{\text{p}}^{2}$ = 0.44, BF_10_ = 385.55, posterior probability > 0.99], revealing larger switch than repeat amplitudes on target 1 at both Pz and Fz sites [*t*_(21)_ = 4.00, *p* < 0.001].

In sum, these results partly mimicked those observed in the conventional ERP waveforms, except that the cP3 window showed main effects for target trial without any interactions with rule updating. In contrast, cLPC_1_ and cLPC_3_ showed different types of interactions between rule updating and target trial, both involving larger amplitudes for switch than repeat target trials (aka “switch positivities”). However, whereas the cLPC_1_ switch positivity was significant only across trials and at the parietal site, the cLPC_3_ switch positivity was significantly enhanced within trials and at both frontal and parietal sites.

#### S cluster

For the sP3 component (Figure 5), the interaction between site and target trial was significant [*F*_(1, 21)_ = 12.39, *p* = 0.002, $\eta_{\text{p}}^{2}$ = 0.37, BF_10_ = 99.43, posterior probability = 0.99], with larger sP3 amplitudes for first than third targets at Fz only [*t*_(21)_ = 3.22, *p* = 0.004]. The main effect of site also reached significance [Pz > Fz; *F*_(1, 21)_ = 16.63, *p* < 0.001, $\eta_{\text{p}}^{2}$ = 0.44, BF_10_ = 371.16, posterior probability > 0.99]. Importantly, no significant effects were found for rule updating.

#### R cluster

For the rP3 component (Figure 6), the three-way interaction between site, rule updating, and target trial was significant [*F*_(1, 21)_ = 13.17, *p* = 0.002, $\eta_{\text{p}}^{2}$ = 0.39, BF_10_ = 128.55, posterior probability = 0.99]. Significant two-way interactions were also found between site and target trial [*F*_(1, 21)_ = 10.66, *p* = 0.004, $\eta_{\text{p}}^{2}$ = 0.34, BF_10_ = 55.48, posterior probability = 0.98] and rule updating and target trial [*F*_(1, 21)_ = 16.38, *p* < 0.001, $\eta_{\text{p}}^{2}$ = 0.44, BF_10_ = 344.98, posterior probability > 0.99]. Main effects for site [Pz > Fz; *F*_(1, 21)_ = 67.68, *p* < 0.001, $\eta_{\text{p}}^{2}$ = 0.76, BF_10_ = 4.60 × 10^6^, posterior probability > 0.99], rule updating [Switch > Repeat; *F*_(1, 21)_ = 10.48, *p* = 0.004, $\eta_{\text{p}}^{2}$ = 0.33, BF_10_ = 52.08, posterior probability = 0.98], and target trial [T1 > T3; *F*_(1, 21)_ = 18.88, *p* < 0.001, $\eta_{\text{p}}^{2}$ = 0.47, BF_10_ = 703.49, posterior probability > 0.99] also reached significance. The *post-hoc* two-way repeated measures ANOVA at Pz revealed larger rP3 amplitudes for switch target 1 than both switch target 3 [*t*_(21)_ = 4.58, *p* < 0.001], and repeat target 1 [*t*_(21)_ = 2.16, *p* = 0.042].

We also examined two conspicuous frontal positivities that were extracted from the R cluster (one pre-rP3 and one post-rP3 at Fz; Figure 6). Two 2 × 2 (rule updating x target trial) repeated measures ANOVAs were conducted at Fz only. For the pre-rP3 positivity, the interaction between rule updating and target trial was significant [*F*_(1, 21)_ = 14.38, *p* = 0.001, $\eta_{\text{p}}^{2}$ = 0.41, BF_10_ = 186.72, posterior probability > 0.99]. *Post-hoc* paired-samples *t*-tests found that frontal pre-rP3 amplitudes for switch target 1 were largest compared to any other target trials (all *p*s < 0.01). Finally, pre-rP3 amplitudes for repeat target 1 were also larger than switch target 3 pre-rP3 amplitudes, [*t*_(21)_ = 3.43, *p* = 0.002].

The frontal post-rP3 positivity also showed a significant interaction between rule updating and target trial [*F*_(1, 21)_ = 18.42, *p* < 0.001, $\eta_{\text{p}}^{2}$ = 0.47, BF_10_ = 614.97, posterior probability > 0.99], and significant main effects for rule updating [*F*_(1, 21)_ = 10.67, *p* = 0.004, $\eta_{\text{p}}^{2}$ = 0.34, BF_10_ = 55.75, posterior probability = 0.98] and target trial [*F*_(1, 21)_ = 5.23, *p* = 0.033, $\eta_{\text{p}}^{2}$ = 0.20, BF_10_ = 6.96, posterior probability = 0.87]. *Post-hoc* paired-samples *t*-tests found that frontal post-rP3 amplitudes were larger for switch target 1 than any other target trials (all *p*s < 0.01). In turn, peak latencies of post-rP3 at Fz were significantly delayed in repeat compared to switch target 1 trials [Mean repeat = 831.1, *SD* = 159.6; Mean switch = 711.6, *SD* = 188.6; *t*_(21)_ = 2.55, *p* = 0.019]. Such differences in post-rP3 peak latencies did not reach significance at Pz.

### Brain-behavior correlations

A series of correlations between RIDE decomposed target P3-like amplitudes and behavioral measures (RTs, accuracy, and switch costs) were conducted. Only Bonferroni-corrected significant correlations that also showed a BF > 3 are presented here. In the C cluster, only mean cLPC_3_ amplitudes at Fz negatively correlated with mean RTs for switch target 1 (*r* = −0.59, *p* = 0.004, BF_10_ = 13.72). In the S cluster, mean sP3 amplitude at Pz negatively correlated with mean RTs in all conditions except for switch target 3 (switch target 1: *r* = −0.53, *p* = 0.012, BF_10_ = 5.05; repeat target 1 *r* = −0.58, *p* = 0.004, BF_10_ = 11.80; repeat target 3 *r* = −0.57, *p* = 0.006, BF_10_ = 9.05). Additionally, mean sP3 amplitude at Pz for repeat target 1 negatively correlated with repeat restart costs (*r* = −0.49, *p* = 0.020, BF_10_ = 3.30). In the R cluster, mean rP3 amplitudes for repeat target 1 negatively correlated with repeat restart costs (*r* = −0.56, *p* = 0.007, BF_10_ = 8.27), and mean amplitudes for the frontal post-P3r peak in repeat target 3 negatively correlated with accuracy (*r* = −0.48, *p* = 0.023, BF_10_ = 2.95). All in all, the correlational analyses suggest that larger RIDE decomposed target P3-like amplitudes in the S, R, and C clusters were associated with faster RTs, higher accuracy and lesser residual costs.

## Discussion

The current study aimed to extend the findings of Barceló and Cooper (2018) by examining the putative role of target P3-like positivities in the reactive control of task-switching using RIDE-decomposed data (i.e., stimulus-locked, response-locked, and central RIDE components). In particular, here we examined the frontoparietal modulations of transient and sustained target P3-like positivities elicited by first and third target trials following transition cues instructing to switch and repeat the ongoing task rule (Figure 3). We expected marked differences between switch and repeat RIDE-decomposed P3-like positivities mostly in the C cluster, based on the assumption that the C cluster best captures cognitive control of higher-order rule updating, which was assumed to be maximally engaged during first target trials following transition cues. Conversely, the S and R clusters were expected to show fewer differences between rule updating and target trial conditions, given that similar sensory stimulation and low-level S-R mappings were employed in all target trials. Whilst the largest expected effects of rule updating and target trial were indeed observed in the C cluster, some task effects in P3-like amplitudes were also apparent in the S and R clusters.

The traditional ERP analyses showed the expected pattern of results in the light of recent findings by Barceló and Cooper (2018), who relied on formal modeling of information transmission between stimuli and responses (see Figure 1B) to identify a whole family of target P3-like positivities whose magnitude and fronto-parietal scalp topographies were dynamically modulated on a trial-by-trial bases as a function of the temporal and task contexts of the eliciting events (cf., Friston et al., 2017; Gratton et al., 2018). That is, target P3 and sustained LPC positivities were found to be largest for the most cognitively demanding target trials (i.e., first switch target trials), and smallest for the cognitively least demanding target trials (i.e., third repeat targets after a transition cue). Similar effects were found for the P3, LPC_1_, and LPC_3_ ERP components in the current study. Specifically, the P3-like positivities elicited by switch and repeat targets are comparable to those reported in previous task-cueing studies where infrequent and temporarily unpredictable switch and repeat cues are interspersed among sequences of temporarily predictable target stimuli. In these task conditions, maximal differences in target P3-like amplitudes following switch vs. repeat cues were observed in the first targets following those cues (Kopp and Lange, 2013), before subsiding or disappearing completely in subsequent trials, as the same task rule was repeatedly rehearsed (cf., Barceló et al., 2008; Barceló and Cooper, 2018). Therefore, the present ERP results concur with the converging efficiency in behavioral responses as participants correctly repeated the same task rule three times following either task cue, as evidenced by the performance asymptotes seen in third target trials (Monsell, 2003).

Hence, it can be assumed that on target 3 the same familiar and well-rehearsed low-order S-R mappings (Figure 1A) had been repeated three times and the protracted effects of cue processing had subsided thus resulting in similar brain and behavioral responses for all task conditions. In contrast, the carry-over effects of cue processing were maximal in first switch target trials, but were also evident in first repeat target trials (cf., Figures 1B, 2). It should be noted, however, that few published task-cueing studies have examined sequential trial-by-trial effects in the amplitude of target P3-like potentials in the first few trials following a cue instructing either to switch or repeat the ongoing S-R mappings (cf., Barceló, 2003; Barceló et al., 2008; Kopp and Lange, 2013; Barceló and Cooper, 2018).

The RIDE results displayed prominent frontal and parietal target P3-like positivities in all three clusters. The largest target LPC was captured by the C cluster, which is the most clearly indicative of higher-order cognitive control operations, such as protracted rule updating and resolution of interference from a previous rule or sensory cue, as will be further discussed below. Interestingly, the S cluster captured not only early sensory processes (90–150 ms), but also later target P3-like activity triggered by the updating of sensory features of stimulation that was modulated across frontoparietal regions by both rule updating and target trial (see Figure 5). In line with the context updating theory of the target P3 (Donchin and Coles, 1988), this implies that updating of the sensory aspects of stimulation does trigger updating of higher-order task-set units (Figure 1A). There also was a visible response-locked target P3-like component (rP3) with maximal parietal scalp distribution, likely reflecting “context updating” elicited by motor or premotor processes associated with trial-by-trial variability in response selection. Overall, these different types of context updating mechanisms all seemed to engage frontal and parietal regions from 300 to 1,200 ms post-stimulus onset, and involved time-varying configuration of neural sources, possibly reflecting either individual differences in the activation of different nodes across a “multiple demand” frontoparietal cortical network for cognitive control (Bledowski et al., 2004; Duncan, 2010), or else, temporarily recurrent frontoparietal activation in the cognitively most demanding task conditions (i.e., first switch target trials).

In the C cluster, the cP3 peak (300–350 ms; at the typical latency of classic P3 potentials) was not modulated by rule updating, implying that the time-variable cP3 was not associated with updating of higher-order task rules. It should be noted, though, that main effects of site and target trial revealed increased mean cP3 amplitudes over both frontal and parietal sites on first compared to third target trials. This effect may possibly reflect cognitive control of “target novelty” to the first target display in the trial run, explaining why cP3 was enhanced at both frontal and parietal regions (Barceló et al., 2008). In sharp contrast, cLPC_1_ and cLPC_3_ amplitudes were both modulated by rule updating, albeit in different ways, with the largest amplitudes for first target trials immediately following a switch cue. Additionally, the cLPC_2_ window only showed a main effect of trial as in the cLPC_1_ and cLPC_3_ windows. These results—larger switch and target trial 1 amplitudes across frontal and parietal regions—suggest that cognitive control associated with the implementation of new low-level S-R links (on repeat trials) and new higher-order rules (on switch trials) were observed over a relatively prolonged 300–1,200 ms time window post-target onset. These findings suggest that cLPC captured several functionally distinct time-varying cognitive control operations resulting in subtly different target P3-like scalp topographies (Barceló and Cooper, 2018; cf., Polich, 2007).

In the S cluster, larger sP3 amplitudes were found on first than third target trials at Fz only, pointing to a frontal, top-down, modulation driven by the first target onset of each trial run, and regardless of cue type. This finding suggests that perceptual context updating of temporarily predictable and familiar target stimuli can also engage frontal regions of the frontoparietal network under conditions of increased cognitive demands (i.e., carryover of interference from a previous task cue; Figure 1B). Thus, the updating of the stimulus context significantly increased sP3 amplitudes on first target trials following a cue, and did so over frontal –but not parietal– regions. This evidence suggests that this specific type of sensory “context updating” recruited more frontal resources in response to *the same* colored Gabor gratings shown in third target trials. Hence, the resulting sP3 component compares to novelty P3/P3a potentials on first target trials only, at a moment when working memory capacity is still overloaded by the processing of the preceding and temporarily unpredictable cueing event. This finding suggests a context-sensitive function of frontoparietal networks, with dynamic trial-by-trial fluctuations in the amount of frontal resources needed for processing the same target gratings when working memory capacity is being taxed by the previous cue relative to subsequent targets in the trial run (Figure 1B; Barceló and Cooper, 2018).

In the R cluster, a parietally distributed rP3 positivity was observed with a similar mean amplitude at Pz for all task conditions, except first switch target trials. Most interestingly, though, both frontal pre-rP3 and post-rP3 positivities were found to be enhanced on targets immediately following a switch cue, likely due to updating of (pre)motor units of sensorimotor S-R mappings in this particular trial. Previous research examining response-locked P3-like positivities is limited, although Gajewski and Falkenstein (2011) found evidence of both frontal and parietal response-locked P3-like peaks occurring 70–80 ms prior to the response. In their study, though, P3-like peaks elicited during task switches were smaller than those elicited during task repetitions. This difference with our study may be attributed to their use of traditional ERP analyses, which makes it plausible that other than purely response-locked processes were inadvertently included in their analyses. More recently, Verleger et al. (2016) found that task difficulty was associated with increased rP3 amplitude at parietal scalp sites occurring approximately 40 ms pre-response, and also that the most cognitively demanding trials (a rare response in a two-choice task) resulted in an additional frontocentral positivity occurring approximately 90 ms pre-response, generally mimicking the rP3 positivities in the current study.

It should be noted that we observed a residual switch benefit (or “repetition cost”), meaning that targets immediately following a repeat cue showed significantly slower RTs than targets immediately following a switch cue (Table 1, Figure 6). When using long cue-target intervals in task-cueing paradigms (>600 ms; Monsell, 2003), absence of local switch costs, or even presence of a paradoxical repetition cost, has often been reported (Schneider and Logan, 2006, 2015; Altmann and Gray, 2008). Actually, absence of a switch cost can be seen as an expected outcome whenever task rule updating is rapidly and fully completed well ahead of target onset (Meiran, 2000). In such situations, switch costs are expected to be reduced to residual costs and, even if these are often larger in switch relative to repeat trials (Monsell, 2017), a paradoxical switch benefit (or “repetition cost”) has been often observed on first repeat trials of intermittent task-cueing studies with long cue-target intervals (Allport and Wylie, 2000; Schneider and Logan, 2006, 2015; Altmann and Gray, 2008; Díaz-Blancat et al., 2018). Various explanations have been proposed for the presence of this residual repetition cost (Monsell, 2017). One is the interfering reactivation of the competing task rule by the first repeat cue that had just been associatively bound to a different task rule in the previous trial run. Another possibility is that switch target trials were processed as the first serial position in a coherent sequence of trials using the same (i.e., color) S-R mapping, which is known to result in a repetition cost on first repetition trials (Schneider and Logan, 2006). These two accounts rely on sequence-level control of sensorimotor associations within a hierarchy of control processes in working memory (Figure 1A; cf., Schneider and Logan, 2015). Moreover, data from Figure 6 suggests there is a strong motor component in this repetition cost, as reflected by the delayed peak latency of the frontal post-rP3 positivity to first repeat target trials. This *post-hoc* hypothesis about a putative role of response-related factors in the residual repetition cost remains an open question for future research.

The current study examined the relatively unexplored sustained switch P3-like positivity to first target trials as originally described by Barceló and Cooper (2018; also see Kopp and Lange, 2013), and investigated the novel hypothesis that this LPC mostly reflects increased cognitive demands due to carryover of interference from the processing of highly informative task cues onto the ensuing first target trials. Overall, the results from the RIDE analyses support this hypothesis. Further, our RIDE analyses show that the traditional dichotomous taxonomy of the P300 complex (i.e., into one frontal P3a and one parietal P3b sub-components; Friedman et al., 2001; Polich, 2007) may be overly simplistic, since the target P3-like positivities elicited by cognitive demands in cued task switching studies are far more nuanced than previously thought. Rather, here we propose that the target P3, including the prolonged LPC, provide a broad conceptual umbrella for a wide family of target P3-like positivities overlying frontal and parietal regions. These target P3-like positivities are clearly distinct functionally and topographically, and they seem to index a variety of context updating operations that require correspondingly distinct types of cognitive control, some of which are stimulus-locked, some response-locked, and some others are latency-variable and best described as intermediate sensorimotor control processes implemented at different hierarchical levels (Figure 1A; cf., Friston et al., 2017). Those manifold P3-like positivities overlay frontoparietal scalp regions –and might reflect activity in various nodes of fronto-parietal networks subserving cognitive control (Bledowski et al., 2004; Duncan, 2010). Furthermore, the present findings concur with the hypothesis that more or less frontal recruitment depends on a dynamic trial-by-trial engagement of cognitive demands (i.e., working memory load) on a trial by trial basis, which is also partly in line with Koechlin and Summerfield's (2007) notion of a rostro-caudal axis for prefrontal executive control (see Figure 1).

The results of the current study have implications for future research in aging populations. Given the general consensus that successful cognitive control recruits a distributed “multiple demand” frontoparietal cortical network (e.g., Duncan, 2010; Niendam et al., 2012), and that a posterior to anterior shift in target P3-like activity is often observed in aging populations under high cognitive demands (Polich, 2007; Enriquez-Geppert and Barceló, 2016), it may be of interest to examine this frontal shift in RIDE-decomposed EEG data, and how this novel technique informs the complex nature of target P3-like potentials in aging populations.

In conclusion, the current study has shown that successful reactive control of target detection is associated with a combination of stimulus-locked, response-locked and temporally variable context-updating operations across frontal and parietal regions of a putative frontoparietal cortical network. These context-updating processes at target onset are likely to temporally overlap, or can even co-occur in time, and they seem to be mediated by antecedent context-updating operations during the cue-target interval (Barceló and Cooper, 2018). Therefore, the context updating theory of the target P3 (Donchin and Coles, 1988), whilst still being generally supported by the present findings, seems to offer an oversimplified picture given the manifold target P3-like positivities observed when different types of context updating operations are examined at different levels in the neural hierarchy of cognitive control (Figure 1A; Miller and Cohen, 2001; Friston et al., 2017).

## Author contributions

CB analyzed the data, prepared the figures and wrote the manuscript. FB conceived and designed the research, and wrote the manuscript. Both authors interpreted and discussed the results.

### Conflict of interest statement

The authors declare that the research was conducted in the absence of any commercial or financial relationships that could be construed as a potential conflict of interest.

## Footnotes

^1^Given the large variability in the latency of target P3 with task complexity, peak latency was not regarded as an *a priori* criterion to differentiate target P3-like subcomponents (Kappenman and Luck, 2012), although switch P3-like positivities are typically sampled from a wider latency window (400–1,000 ms) than the regular target P3 (300–400 ms) elicited in simple target detection tasks.

^2^Note that in the study by Barceló and Cooper (2018), all targets conveyed, on average, the same amount of information for response selection, whereas the relatively infrequent switch cues –that were not to be overtly responded to– conveyed the largest amount of information for response selection regarding the ensuing first target trial. What seemed less intuitively obvious though, was that the infrequent repeat cues also overloaded working memory capacity, albeit to a lesser extent than the switch cues (Figure 1B).

## Abbreviations

BF: Bayes factor
ERP: event-related potential
LPC: late positive complex
RIDE: residue iteration decomposition
RT: reaction time.
