# Supplementary material for: Functional Dissociation of Latency-Variable, Stimulus- and Response-Locked Target P3 Sub-components in Task-Switching
Source: Front Hum Neurosci. 2018 Feb 20;12:60. doi: 10.3389/fnhum.2018.00060 (PMC5826240; doi:10.3389/fnhum.2018.00060)
Supplement: Supplementary file 1 [file Table1.PDF]

Supplementary Material

Functional dissociation of latency-variable, stimulus- and response-locked target P3 sub-components in task-switching

Christopher R. Brydges[1,2*] and Francisco Barceló[1*]

*** Correspondence:** christopherbrydges@gmail.com; f.barcelo@uib.es

Information theoretic estimations of sensory, motor, and sensorimotor control at two levels of a putative hierarchy of cognitive control for the Switch task (Figs. 1A,B; cf., Attneave, 1959; Barceló & Cooper, 2018; Koechlin & Summerfield, 2007; Miller, 1956; Miller & Cohen, 2001).

Stimulus Entropy: $H(s_i) = -\Sigma_{i=1}^{6} p(s_i) \cdot \log_2 p(s_i)$

Table S1. Estimates of stimulus entropy and surprise. Visual displays, stimulus codes, stimulus entropies, mean stimulus probabilities, and information surprise for six Gabor stimuli used in the Switch task.

Gabors	S^1	$H(s)$	$p(s)$	$-\log_2 \cdot p(s)$
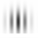	s_1	0.29	0.08	3.64
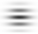	s_2	0.29	0.08	3.64
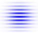	s_3	0.47	0.21	2.25
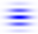	s_4	0.47	0.21	2.25
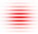	s_5	0.47	0.21	2.25
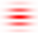	s_6	0.47	0.21	2.25
	$\Sigma H(s_i)=$	2.46		

Response Entropy: $H(r_j) = -\Sigma_0^j p(r_j) \cdot \log_2 p(r_j)$

The Switch task required two-button responses (r_1, r_2) and the absence of response (r_0) to the gray gratings. The response set can be defined as $\boldsymbol{R} = \{r_0, r_1, r_2\}$, and estimates of response entropy and surprise are shown in Table S2.

Table S2. Estimates of response entropy and surprise. Response codes, response entropies, mean response probabilities, and information surprise for the set of three possible responses in the Switch task.

R	H(r)	p(r)	$-\log_2 \cdot p(r)$
r_0	0.43	0.16	2.60
r_1	0.53	0.42	1.26
r_2	0.53	0.42	1.26
$\Sigma H(r_i)=$	1.49		

[1] For simplicity, these estimates assume a stimulus set $\{S\}$ with only six stimuli, regardless of the fact that each Gabor grating was randomly displayed either to the left or the right visual hemifields.

Low-order Sensorimotor (S-R) Information:

$$I(s_i, r_j) = \log_2 p(s_i, r_j) - \log_2 p(s_i) - \log_2 p(r_j)$$

Information transmitted from stimuli to responses, $I(s_i, r_j)$, is derived from the notion of mutual information between sets of stimuli $\{S\}$ and responses $\{R\}$ (Attneave, 1959):

$$I(S;R) = \sum_i \sum_j p(s_i, r_j) \log \frac{p(s_i, r_j)}{p(s_i) p(r_j)}$$

The Switch task required two-choice responses (r_1, r_2) to categorize four colored Gabor gratings (s_3, s_4, s_5, s_6). Estimates of low-level sensorimotor control for the relevant task-set units (s_i-r_j) when sorting either by the Color or spatial Frequency rules are shown in Table S3.

Table S3. Estimates of transmitted low-order sensorimotor (s-r) information [2]. Codes for hypothetical unitary S-R pathways, mean stimulus and response probabilities, joint s-r probabilities, and transmitted low-order s-r information.

S-R	$p(s_i)$	$p(r_j)$	$p(s_i, r_j)$	$I(s_i, r_j)$
s_1-r_0	0.08	0.16	0.08	2.61
s_2-r_0	0.08	0.16	0.08	2.61
s_3-r_1	0.21	0.42	0.21	1.26
s_4-r_1	0.21	0.42	0.21	1.26
s_5-r_2	0.21	0.42	0.21	1.26
s_6-r_2	0.21	0.42	0.21	1.26
				$\Sigma(s_i r_j)$= 10.26

High-order Sensorimotor (Episodic) Information:

$$I(s_i, ts_k) = \log_2 p(s_i, ts_k) - \log_2 p(s_i) - \log_2 p(ts_k)$$

The switch task required access to episodic task-set (ts_1) information on just 8% of all trials (s_1-ts_1), while no task-set access (ts_0) was required in all remaining trials. Thus, the transmitted information between sensory stimuli and access to episodic memories in the switch task can be estimated as shown in Table S4.

[2] Sensorimotor information in the switch task was slightly larger for those color gratings that afforded bivalent responses (i.e., different buttons for either rule). For simplicity this additional source of contextual information was not included here.

Table S4. Estimates of transmitted high-order sensorimotor information between stimulus units and task-set units. Codes for hypothetical links between stimulus and task-set units, mean stimulus and task-set probabilities, joint *s-ts* probabilities, and transmitted episodic information for the switch task.

S-TS	$p(s_i)$	$p(ts_k)$	$p(s_i, ts_k)$	$I(s_i, ts_k)$
s_1-ts_1	0.08	0.08	0.08	3.61
s_2-ts_0	0.21	0.92	0.21	0.12
s_3-ts_0	0.21	0.92	0.21	0.12
s_4-ts_0	0.21	0.92	0.21	0.12
s_5-ts_0	0.21	0.92	0.21	0.12
s_6-ts_0	0.21	0.92	0.21	0.12
				$\Sigma(s_i ts_k)$= 4.21

Estimates of overall transmitted S-R information for each task stimulus are given in Table S5 (see Figs. 1A,B), and were computed as summed information across two levels in the putative hierarchy of sensorimotor information processing (i.e., switch cues, s_1 = 2.61 + 3.61= 6.22 bits; repeat cues, s_2 = 2.61 + 0.12= 2.73 bits; colored gratings, s_3 = 1.26 + 0.12= 1.38 bits).

Table S5. Numerical estimates of transmitted information in Figure 1B (in bits).

	s_1	s_2	s_3	s_4	s_5	s_6
Switch task	6.22	2.73	1.38	1.38	1.38	1.38

References

Attneave, F. (1959). *Applications of information theory to psychology: A summary of basic concepts, methods and results*. New York: Holt, Rinehart and Winston.

Barceló, F., & Cooper, P. (2018). An information theory account of late frontoparietal ERP positivities in cognitive control. *Psychophysiology*, in press.

Koechlin, E., & Summerfield, C. (2007). An information theoretical approach to prefrontal executive function. *Trends in Cognitive Sciences*, *11*, 229-235.

Miller, G.A. (1956). The magical number seven plus or minus two: some limits on our capacity for processing information. *Psychological Review*, *63*, 81-97.

Miller, E. K., & Cohen, J. D. (2001). An integrative theory of prefrontal cortex function. *Annual Review of Neuroscience*, *24*, 167-202.
